# Radiologically isolated syndrome and the possibility of preclinical disease activity in aquaporin-4 antibody NMOSD

**DOI:** 10.1177/13524585221085732

**Published:** 2022-03-25

**Authors:** Dalia L Rotstein

**Affiliations:** Department of Medicine, University of Toronto, Toronto, ON, Canada; MS Clinic, St. Michael’s Hospital, Toronto, ON, Canada

**Keywords:** NMOSD, aquaporin-4 antibody, radiologically isolated syndrome

Neuromyelitis Optica Spectrum Disorder (NMOSD) is a frequently devastating inflammatory disease of the central nervous system. Diagnosis is based on serum testing for the aquaporin-4 (AQP4) antibody in conjunction with at least one typical clinical feature.^1^ However, positive AQP4 serology has been reported many years in advance of clinical presentation^2^ which implies pathogenic mechanisms at work much earlier than recorded onset – and, potentially, a missed opportunity for early identification and intervention.

In this issue of *Multiple Sclerosis Journal*, Abdel-Mannen et al.^3^ present a case of radiologically isolated NMOSD in an asymptomatic patient. Disc edema was picked up on routine eye exam which led to magnetic resonance imaging, revealing optic nerve enhancement, and positive serum AQP4-antibody testing. The patient was treated with pulsed high-dose corticosteroids, then rituximab, and remained asymptomatic. We cannot know if and when she would have developed symptoms in the absence of such therapy, but this case underscores the full spectrum of AQP4 + antibody disease activity, including the possibility, albeit rare, of very mild or asymptomatic cases.

As with multiple sclerosis, whether we should treat ‘radiologically isolated’ findings or, in the context of NMOSD a positive antibody, without concomitant clinical symptoms requires further consideration and follow-up. On one hand, the well-documented, very high specificity of the cell-based AQP4-antibody assay might argue for early and sustained immunotherapy.^4^ However, the need for lifelong immunosuppression for confirmed AQP4 + NMOSD carries its own burdens, and pathogenicity of the antibody may depend on breach of the blood-brain barrier and ongoing permeability.^5^ If, in the future, other asymptomatic patients are identified and followed off therapy, we could learn more about predictive biomarkers, pathogenetic mechanisms and clinical evolution from the earliest stages of AQP4 + NMOSD, although patients would have to be very closely watched to allow for rapid intervention if symptoms manifest. These findings could facilitate earlier recognition of NMOSD in other clinical scenarios as well, and ultimately more timely application of immunosuppressive therapy and novel immune tolerance approaches.
